# Genome-Wide Association Study Reveals Novel Candidate Genes Influencing Semen Traits in Landrace Pigs

**DOI:** 10.3390/ani14131839

**Published:** 2024-06-21

**Authors:** Zhanwei Zhuang, Kebiao Li, Kai Yang, Guangxiong Gao, Zhili Li, Xiaoping Zhu, Yunxiang Zhao

**Affiliations:** 1 College of Animal Science and Technology, Guangxi University, Nanning 530004, China; zwzhuang@gxu.edu.cn; 2 School of Life Sciences and Engineering, Foshan University, Foshan 528231, China; bb973900622@163.com (K.L.); 2112059007@stu.fosu.edu.cn (K.Y.); 18312090361@163.com (G.G.); pinganzhili@163.com (Z.L.); zhuxiaoping@fosu.edu.cn (X.Z.); 3 Guangxi Yangxiang Co., Ltd., Guigang 537100, China

**Keywords:** pig breeding, boar semen traits, genetic architecture, SNP

## Abstract

**Simple Summary:**

Improving the reproductive performance of boars is a major goal in pig-breeding programmes. Semen traits are significantly associated with the reproductive performance of boars, and thus, identifying single nucleotide polymorphisms (SNPs) and the genes affecting semen traits are important. Genome-wide association study have been widely used to detect significant SNPs associated with economically important traits in pigs. The results of our study can be used in genomic selection models to facilitate future breeding programs aimed at improving reproductive traits in boars.

**Abstract:**

Artificial insemination plays a crucial role in pig production, particularly in enhancing the genetic potential of elite boars. To accelerate genetic progress for semen traits in pigs, it is vital to understand and identify the underlying genetic markers associated with desirable traits. Herein, we genotyped 1238 Landrace boars with GeneSeek Porcine SNP50 K Bead chip and conducted genome-wide association studies to identify genetic regions and candidate genes associated with 12 semen traits. Our study identified 38 SNPs associated with the analyzed 12 semen traits. Furthermore, we identified several promising candidate genes, including *HIBADH*, *DLG1*, *MED1*, *APAF1*, *MGST3*, *MTG2*, and *ZP4*. These candidate genes have the potential function to facilitate the breeding of boars with improved semen traits. By further investigating and understanding the roles of these genes, we can develop more effective breeding strategies that contribute to the overall enhancement of pig production. The results of our study provide valuable insights for the pig-breeding industry and support ongoing research efforts to optimize genetic selection for superior semen traits.

## 1. Introduction

Artificial insemination (AI) is a widely used technology in swine breeding that enables the efficient dissemination of improved genetics from elite boars to commercial pig populations. To achieve successful AI outcomes, it is essential to produce high-quality semen characterized by favorable genetic traits, good sperm motility, and a high number of sperm cells per ejaculate. However, the semen quality of boars is affected by various factors, such as nutrition, disease, the interval between the ejaculate seasons, and age [1]. In addition, genetics plays an important role in semen quality; semen traits are known to have low to moderate heritability [2,3].

Currently, there are several approaches to evaluate semen quality [4], including measuring semen volume, concentration, motility, progressive motility, and the total proportion of sperm morphological abnormalities. These approaches are crucial for commercial boar stations to maintain the consistency and stability of boar semen quality. In recent years, with advances in high-throughput genotyping and molecular techniques, there has been growing interest in understanding the molecular processes and genetic mechanisms that influence semen traits. Although several genes and markers associated with pig-semen traits were identified and described in previous studies [5,6], few studies have extensively analyzed large datasets to identify novel quantitative trait loci (QTL) and provide deeper insights into the genes controlling the semen traits of boars. This is likely due to the genetic complexity of sperm cell production and maturation. 

Genome-wide association studies (GWAS) are commonly used to identify single-nucleotide polymorphisms (SNPs) associated with traits with major effects [7]. To enhance these traits, traditional selection methods must be supplemented with marker-assisted selection. This can be achieved by identifying the genotypes of reproductive traits and their relationships by determining the associated polymorphisms and phylogenetic relationships.

Previous studies have reported on the genetic regions and/or candidate genes associated with boar semen traits [8,9,10], contributing to our understanding of the genetic architecture of swine semen traits. These studies varied in terms of the genetic background, number of boars, and density of the genetic marker panels used. Therefore, the objective of the present study was to identify the QTL regions and candidate genes associated with semen quality in the Landrace boar population using GWAS. 

## 2. Materials and Methods

### 2.1. Phenotypic and Pedigree Data

We collected 225,468 semen samples from 2059 Landrace boars (including 1238 genotyped pigs) in three locations (Tiantishan, Daling, and Gongzhutun) operated by Guangxi Yangxiang Co., Ltd. (Guigang, China) from 2016 to 2021. The 2059 boars both had phenotypic data and pedigree information and were used to acquire estimated breeding values (EBV). The age of the boars ranged from 5 to 71 months. All boars were raised in hog houses with the temperature, humidity, and wind speed automatically controlled. Each boar was allocated to a single pen with approximately 7 m^2^ of space and fed a specialized diet. Semen traits, including semen density (DEN), semen motility (MOT), and percentage of abnormal sperms (ABN) were directly measured using computer-aided sperm analysis. The semen volume (VOL) was measured by weight assuming that 1 mL = 1 g. Other semen traits such as total sperm number (TSN) and functional sperm number (FSN) were calculated using the following formulae [11]:

TSN=VOL×DEN

FSN=TSN×MOT×(1−ABN)

To ensure quality, semen data were retained only if they met the following criteria: VOL range of 30–800 mL, DEN range of 0.01–10 × 10^8^/mL, MOT range of 10–99%, and ABN range of 0–90%.

We calculated the coefficient of variation (CV) for semen data collected in the same year and season to measure the degree of variation in boar semen data as follows:

CV=σ/μ

where CV was the coefficient of variation of the semen traits of Landrace, σ was the standard deviation of the semen traits of Landrace, and μ was the average of the semen traits of Landrace.

### 2.2. Genotypic Data

Total DNA was extracted from 1238 boars using genome extraction kits (Wuhan NanoMagBio Technology Co., Ltd., Wuhan, China), according to manufacturer’s instructions. Boars were genotyped using the GeneSeek Porcine SNP50 K Bead chip data (Neogen, Lansing, MI, USA), which contains 50,703 SNPs. The SNP positions were remapped to the *Sus scrofa* 11.1 reference genome using the genome remapping procedure available from the NCBI (National Center for Biotechnology Information). Autosomal SNPs were filtered using PLINK v1.9, as previously described [12], based on the following quality control criteria: individual call rate ≥90%, SNP call rate ≥90%, minor allele frequency ≥0.01, and Hardy–Weinberg equilibrium *p*-value ≥ 10^−6^. After quality control, 1238 boars and 43,876 SNPs were retained for further analysis. Missing genotypes were imputed using the Beagle software (version 4.1), as previously described [13]. Subsequently, the imputed SNPs were subjected to another round of quality control using the same criteria mentioned above in PLINK v1.9.

### 2.3. Statistical Model

Variance components were estimated using DMUAI, which were then used in the DMU to predict estimated breeding values (EBV). The multi-trait animal model for PBLUP (Pedigree Best Linear Unbiased Prediction) was as follows:

Y=Xb+Za+Wp+Age+Intv+e

where Y was the vector of phenotypic values; b was the vector of fixed effects, including center-year-season (levels are shown in Figure S1); a~N(0, Aσ_a_^2^) was the vector of additive genetic effects of the boar; p(0, Iσ_p_^2^) was the vector of the permanent environmental effect of the boar; Z and W were design matrices for a and p, respectively; age represents the age of the boar; Intv was the interval between the present and previous semen collection time points; and e(0, Iσ_e_^2^) was the residual effect. It was assumed that ~N(0, Aσ_a_^2^), p(0, Iσ_p_^2^), and e(0, Iσ_e_^2^), where σ_a_^2^, σ_p_^2^ and σ_e_^2^ were additive genetic, permanent environmental, and residual variances, respectively. A was a matrix that combined pedigree information and I was an identity matrix. The pedigree information used in this study encompassed a pedigree of 5284 pigs across three generations.

We then obtained deregressed EBVs (DEBVs) for the 1238 boars. DEBVs were calculated as follows [14,15]:

DEBV=PA+(EBV−PA)/REL

$$REL=1-std/\sigma_{a}^{2}$$

where DEBV was the deregressed estimated breeding value of each boar, PA represents the parental average, EBV was the estimated breeding value, REL was the reliability of each boar, and std was the standard error of the EBV of each boar. σ_a_^2^ was the additive effect variance for the relative traits.

We performed GWAS using the Circulating Probability Unification (FarmCPU) model with rMVP software (version 1.0.8) as previously described [16] in 1238 genotyped pigs. This model used fixed and random effects to control for false negatives simultaneously. The model was expressed as follows:

$$Y=Tw_{i}+P_{j}q_{j}+m_{k}h_{k}+e$$

where Y was the vector of DEBV; T was a matrix of fixed effects, including the top three principal components with the corresponding effect; P_j_ was the genotype matrix of j pseudo-quantitative trait nucleotides (QTNs), which were used as a fixed effect and q_j_ was the corresponding effect; m_k_ was a vector of genotypes for the kth marker to be tested and h_k_ was the response effect; and e was the residual effect vector with distribution e~(0, Iσ_e_^2^), where σ_e_^2^ represented the residual variance. The random-effects model was used to select the most appropriate pseudo-QTNs.

The genome-wide significance threshold was set at *p* < 0.05/N, where N was the number of qualified SNPs. In this study, N was 43,876 and the threshold was set to 1.14 × 10^−6^. Phenotypic and genetic correlations among the semen traits were calculated using “asreml” package in R-4.0.4 (www.r-project.org, accessed on 23 December 2023) statistical environment and were used to determine whether there were associations between the GWAS results.

### 2.4. Annotation of Candidate Genes

We identified potential candidate genes within 500 kb up- and downstream of genome-wide significant SNPs in the *Sus scrofa* 11.1 genome from the Ensembl database. Candidate genes were selected for traits according to their biological function using NCBI and Genecards database.

## 3. Results

### 3.1. Phenotypic Data Analysis and Heritability Estimates

We collected 225,468 semen samples from 2059 boars and calculated the VOL, DEN, MOT, ABN, TSN, and FSN for each sample. The descriptive statistics of the 12 semen traits are listed in Table 1. In brief, the coefficient of variation (C.V) value of the 11 analyzed semen traits ranged from 25.07% (CV_VOL_) to 89.01% (ABN), thereby indicating the potential genetic improvement space for the traits. Table S1 shows the data distribution of DEBV. Table S2 shows the estimates of the variance components of the semen traits. The genomic heritability estimates for VOL, DEN, MOT, ABN, TSN, FSN, CV_VOL_, CV_DEN_, CV_MOT_, CV_ABN_, CV_TSN_, and CV_FSN_ were 0.20, 0.17, 0.23, 0.24, 0.11, 0.15, 0.017, 0.003, 0.001, 0.011, 0.002, and 0.044, respectively.

### 3.2. The Genetic and Phenotypic Correlation Coefficients of Semen Traits in Boars

Table 2 shows the correlation coefficients of the semen traits in boars. The correlation coefficient between the traits was −0.02 to 0.99. Significant negative correlations were found between VOL and DEN (−0.62), VOL and ABN (−0.12), DEN and ABN (−0.29), MOT and ABN (−0.87), TSN and ABN (−0.62), and FSN and ABN (−0.76). Positive correlations were found between VOL and MOT (0.06), VOL and TSN (0.27), VOL and FSN (0.26), DEN and MOT (0.38), DEN and TSN (0.53), DEN and FSN (0.53), MOT and TSN (0.73), MOT and FSN (0.81), and TSN and FSN (0.98). The *p*-value of the correlation between all the above traits was less than 0.05.

Concerning the phenotypic correlation of the semen traits, positive correlations were observed between VOL and TSN (0.38), VOL and FSN (0.38), DEN and MOT (0.16), DEN and ABN (0.08), DEN and TSN (0.57), DEN and FSN (0.55), MOT and TSN (0.31), MOT and FSN (0.36), and TSN and FSN (0.99). There was a negative correlation between semen traits, such as VOL and DEN (−0.43), VOL and MOT (−0.02), VOL and ABN (−0.02), ABN and MOT (−0.47), ABN and TSN (−0.02), and ABN and FSN (−0.16).

### 3.3. GWAS for Semen Traits and Candidate Gene Searching

**Figure 1:**
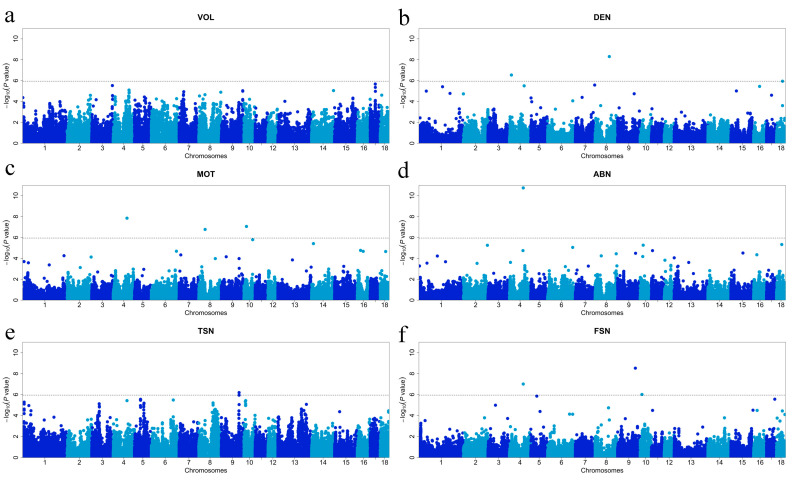
Manhattan plots of the GWAS for semen traits in boars. (**a**) GWAS for VOL; (**b**) GWAS for DEN; (**c**) GWAS for MOT; (**d**) GWAS for ABN; (**e**) GWAS for TSN; (**f**) GWAS for FSN.

**Figure 2:**
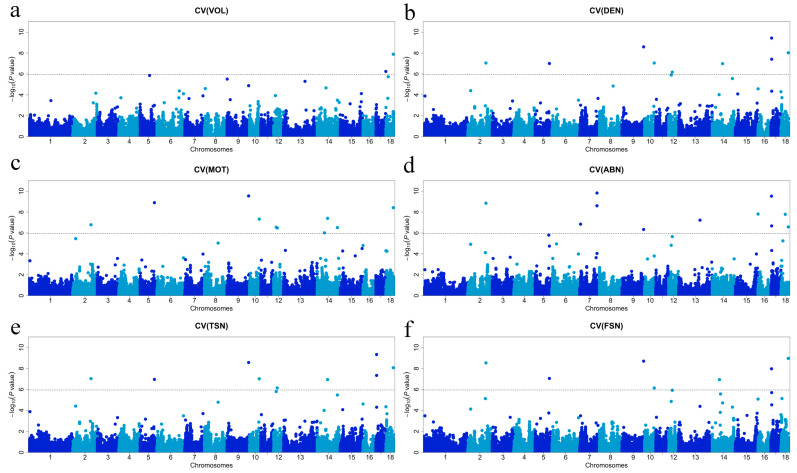
Manhattan plots of the GWAS for Semen traits in boars. (**a**) GWAS for CV_VOL_; (**b**) GWAS for CV_DEN_; (**c**) GWAS for CV_MOT_; (**d**) GWAS for CV_ABN_; (**e**) GWAS for CV_TSN_; (**f**) GWAS for CV_FSN_.

The results of the GWAS for semen traits are shown in Figure 1 and Figure 2 and Table 3. There were no significant SNPs to be associated with VOL. Two significant SNPs associated with DEN were identified, MARC0085656 and ALGA0048589, located on chromosomes 4 and 8, respectively. There were three significant SNPs associated with MOT, ALGA0026338, H3GA0024811, and ASGA0101355, located on chromosomes 4, 8, and 10, respectively. There was one significant SNP associated with ABN, ALGA0026338, located on chromosome 4. One significant SNP associated with TSN was found, WU_10.2_9_120884225, located on chromosome 9. There were three significant SNPs associated with FSN identified, ALGA0026338, WU_10.2_9_120884225, and H3GA0029253, located on chromosomes 4, 9, and 10, respectively. Two significant SNPs associated with CV_VOL_ were found, WU_10.2_17_69108942 and ASGA0080065, located at chromosomes 17 and 18, respectively. Nine SNPs significantly associated with DEN were identified: MARC0067122, ASGA0094241, ALGA0055832, WU_10.2_10_65681460, MARC0113309, DRGA0013964, WU_10.2_17_5093088, ALGA0110656, and ASGA0080065. Ten SNPs significantly associated with MOT were identified: MARC0067122, ASGA0094241, ALGA0055832, WU_10.2_10_65681460, MARC0097207, MARC0113309, ALGA0077301, DRGA0013964, WU_10.2_14_135493445, and ASGA0080065. Ten SNPs significantly associated with ABN were found including MARC0067122, WU_10.2_7_113446920, MARC0029691, ALGA0055832, ASGA0058857, WU_10.2_16_238186, WU_10.2_17_4199456, ALGA0110656, and Affx-115137014. Nine significant TSN-associated SNPs were identified: MARC0067122, ASGA0094241, ALGA0055832, WU_10.2_10_65681460, MARC0113309, DRGA0013964, WU_10.2_17_5093088, ALGA0110656, and ASGA0080065. Seven SNPs significantly associated with FSN were identified: MARC0067122, ASGA0094241, ALGA0055832, WU_10.2_10_65681460, ALGA0077301, WU_10.2_17_4199456, and ASGA0080065. Candidate genes around the identified SNPs were searched and listed in Table 3.

## 4. Discussion

In recent years, there have been an increasing number of studies on genomic regions affecting boar semen traits, owing to advances in molecular and genotyping techniques, statistical methods, and the application of GWAS [9,10]. In this study, we performed a GWAS to identify QTL regions and candidate genes associated with semen quality in a Landrace boar population. A total of 38 SNPs and 13 genes were considered to be candidate markers associated with semen traits.

The *HIBADH* (3-hydroxyisobutyrate dehydrogenase) gene encodes mitochondrial 3-hydroxyisobutyrate dehydrogenase and may be associated with certain semen traits in men, such as sperm motility [17]. In cattle, *HIADBH* was reported to be related to low sperm vitality through whole genome association analysis [18]. Our results suggest *HIBADH,* located at the SNP ASGA0080065 on chromosome 18, is significantly correlated with six semen traits: CV_VOL_, CV_DEN_, CV_MOT_, CV_ABN_, CV_TSN_, and CV_FSN_. However, the underlying mechanism whereby *HIBADH* affects semen traits is not well understood; further research is required to comprehensively elucidate this association.

The *DLG1* (discs large MAGUK scaffold protein 1) gene, located at SNP ASGA0058857 on chromosome 13, significantly correlated with CV_VOL_ and CV_ABN_. *DLG1* was shown to be related to litter size via the Hippo signaling pathway, which plays a key role in mechanical transduction in Pelibuey sheep [19]. DLG1 is a scaffold protein that participates in controlling key cellular processes, such as polarity, proliferation, and migration, by interacting with various cell partners [20]. *DLG1* is highly expressed in oocytes and granulosa cells [21] and encodes MAGUK protein family members involved in epithelial cell polarization in mice [22].

The *MED1* (mediator complex subunit 1) gene (also known as *NR2C2*) encodes a protein called mediator subunit 1, which is a component of the transcriptional mediator complex and was significantly associated with CV_DEN_, CV_MOT_, CV_ABN_, CV_TSN_, and CV_FSN_ in the current study. MED1 is thought to act as a bridge between transcriptional activators and the RNA polymerase II complex, thereby facilitating the transcription of target genes. MED1 is involved in the regulation of various biological processes, including development, cell proliferation, and apoptosis [23,24]. Bovo et al. [25] found that *MED1* was related to calcium ion concentration in the serum of large white pigs. Calcium ions play a role in sperm motility and fertilization and may be involved in regulating the contractile activity of smooth muscle cells in the vas deferens and the duct that carries sperm from the testes to the urethra [26]. Calcium ions may also play a role in the acrosome reaction by which the sperm acrosome (a structure containing hydrolytic enzymes) is released during fertilization [27]. 

The *APAF1* (apoptotic peptidase activating factor 1) gene, located in the WU_10.2_5_89558832 SNP on chromosome 5, was found to be significantly correlated with CV_ABN_. *APAF1* is a central component of the apoptosome, a multi-protein complex that activates procaspase-9 after cytochrome c is released from the mitochondria in the intrinsic pathway of apoptosis [28]. A C/T mutation in this gene affects the success rate of sperm fertilization and the mummy rate in dairy cows [29]. 

The *MGST3* (microsomal glutathione S-transferase 3) gene, located in SNP ALGA0026338 of chromosome 4, was found to be significantly correlated with three semen traits: MOT, ABN, and FSN. *MGST3* participates in the synthesis of prostaglandins that are involved in cell differentiation and apoptosis [30]. 

The *EFNA5* (ephrin A5) gene, located at SNP MARC0067122 on chromosome 2, was identified as a candidate gene correlated with five semen traits: CV_DEN_, CV_MOT_, CV_ABN_, CV_TSN_, and CV_FSN_. The loss of *EFNA5* in female mice results in subfertility, implying that Eph–ephrin signaling may also play a previously unidentified role in fertility regulation in women. *EFNA5* is highly expressed in cancerous prostate tissues, suggesting that *EFNA5* may participate in prostate carcinogenesis [31]. The prostate is the largest organ that secretes prostate fluid, which is an important component of semen. 

The *SS18L1* (SS18L1 subunit of BAF chromatin remodeling complex) gene on chromosome 17 was found to have a significant relationship with CV_VOL_ in our study. The methylation level of *SS18L1* in sperm from low-fertility buffaloes was found to be significantly higher than that in sperm from high-fertility buffaloes [32]. This further suggests that *SS18L1* could be a candidate gene for influencing semen traits. *SS18L1* is significantly related to disease resistance and heat tolerance in the Dehong humped cattle population through genome-wide selection [33]. This suggests that the quality of boar semen may not only be affected by genetics but also by the external environment, including disease, temperature, humidity, and nutrition. Boars with stronger disease and heat resistance may be less affected by these factors in terms of semen quality. 

AKR1B1 (aldo-keto reductase family 1 member B), a protein present in bovine epididymal spermatozoa, which is found exclusively in the detergent-soluble fractions of detergent-resistant membranes [34], may be compartmentalized in the lumen of the epididymosomes but not at the membrane surface [35]. The intracellular localization of AKR1B1 may be involved in the accumulation of sorbitol within the cytoplasm, which confers protection to epididymal spermatozoa against hypertonic conditions and enhances sperm survival during epididymal transit and storage [36,37]. Significant differences were found in the expression of *AKR1B1* between blastocysts of different qualities [38,39]. 

*ZP4* (zona pellucida (ZP) glycoprotein 4) was suggested to play important roles in species-specific sperm–egg binding, preventing polyspermy, ZP-induced acrosome reactions, and protecting the embryo [40]. Human *ZP4* alone is insufficient to support the binding of human sperm to the ZP in transgenic mice, suggesting that other zona proteins may also play a role in human gamete recognition [41,42]. *ZP4* was shown to induce the acrosome reaction and improve the efficiency of in vitro porcine fertilization [43]; however, *ZP4* is probably dispensable for female fertility [44]. 

In the present study, other candidate genes associated with semen traits in Landrace pigs were identified, such as *HSD17B12* (hydroxysteroid 17-beta dehydrogenase 12), *HABP2* (hyaluronan binding protein 2), *CTNND2* (catenin delta 2), and *MTG2* (mitochondrial ribosome-associated GTPase 2); however, information on their function in relation to semen traits is limited.

## 5. Conclusions

38 SNPs were found to be associated with 12 semen traits in Landrace pigs using GWAS. Of the SNPs associated with the candidate genes, *ZP4* may be worth further investigation. These findings provide valuable insights into future molecular breeding of boar semen traits in the context of genomic selection by allowing the identified SNPs to be assigned with higher weights in models.

## Figures and Tables

**Table 1:** Descriptive statistics of semen traits.

Trait	N1	N2	Average	Mean	S.D	Min	Mam	C.V (%)
VOL, mL	2059	225,468	109.50	207.18	78.26	31.00	650.00	37.77
DEN, 10^8^/mL	2059	225,468	109.50	3.73	1.72	0.03	10	46.11
MOT, %	2059	225,468	109.50	88.72	7.16	11.00	99.80	8.07
ABN, %	2059	225,468	109.50	7.28	6.48	0.00	96.00	89.01
TSN, 10^8^	2059	225,468	109.50	636.45	274.63	0.80	2313.90	43.15
FSN, 10^8^	2059	225,468	109.50	590.36	255.73	0.80	2198.21	43.32
CV_VOL_, %	2059	15,992	7.76	29.00	7.27	3.06	82.03	25.07
CV_DEN_, %	2059	15,992	7.76	36.42	10.32	10.00	209.44	28.34
CV_MOT_, %	2059	15,992	7.76	7.03	5.52	0.55	62.08	78.52
CV_ABN_, %	2059	15,992	7.76	55.82	18.86	11.53	264.58	33.79
CV_TSN_, %	2059	15,992	7.76	37.00	12.62	8.36	210.39	34.11
CV_FSN_, %	2059	15,992	7.76	37.66	13.44	10.69	220.22	35.69

N1: the number of boars; N2: the records of semen data; Average: average number of samples per boar; S.D: Standard deviation; Min: minimum; Max: maximum; C.V: coefficient of variation.

**Table 2:** Correlation coefficients of semen traits in the boars.

	VOL	DEN	MOT	ABN	TSN	FSN
VOL		−0.43	−0.02	−0.02	0.38	0.38
DEN	−0.62 (0.07)		0.16	0.08	0.57	0.55
MOT	0.06 (0.11)	0.38 (0.10)		−0.47	0.31	0.36
ABN	−0.12 (0.09)	−0.29 (0.09)	−0.87 (0.03)		−0.02	−0.16
TSN	0.27 (0.11)	0.53 (0.08)	0.73 (0.07)	−0.62 (0.08)		0.99
FSN	0.26 (0.10)	0.53 (0.08)	0.81 (0.05)	−0.76 (0.05)	0.98 (0.01)	

Upper diagonal shows phenotypic correlation; lower shows genetic correlation, correlation before parentheses, standard error within parentheses.

**Table 3:** Candidate genes for semen traits in pigs.

SNP	Traits	Chromosome	Position	Located Gene	Flanking Genes
ALGA0107690	CV_MOT_, CV_ABN_	2	18,451,516	-	ALKBH3/HSD17B12
MARC0067122	CV_DEN_, CV_MOT_, CV_ABN_, CV_TSN_, CV_FSN_	2	112,667,955	EFNA5	-/FBXL17
MARC0085656	DEN	4	12,149,395	-	-/MYC
ALGA0026338	MOT, ABN, FSN	4	85,073,958	MGST3	TMCO1/LRRC52
MARC0066013	CV_VOL_	5	59,098,986	-	GRIN2B/EMP1
WU_10.2_5_89558832	CV_ABN_	5	85,236,461	APAF1	ANKS1B/IKBIP
ASGA0094241	CV_DEN_, CV_MOT_, CV_ABN_, CV_TSN_, CV_FSN_	5	88,898,348	CEP83	TMCC3/PLXNC1
WU_10.2_6_25519992	CV_ABN_	6	28,217,706	CTCF	AGRP/CARMIL2
ASGA0030861	CV_ABN_	7	5,812,005	-	SLC35B3/-
WU_10.2_7_113446920	CV_ABN_	7	106,992,267	-	-/-
MARC0029691	CV_ABN_, CV_FSN_	7	107,017,794	-	-/-
H3GA0024811	MOT	8	39,096,198	-	DCUN1D4/SGCB
MARC0054558	CV_DEN_, CV_MOT_, CV_TSN_	8	84,708,131	-	GAB1/-
ALGA0048589	DEN	8	88,060,364	-	NOCT/-
ALGA0118936	CV_VOL_	9	1,823,736	-	-/OVCH2
WU_10.2_9_120884225	TSN, FSN	9	109,865,752	-	CUL1/-
ALGA0055832	CV_VOL_, CV_DEN_, CV_MOT_, CV_ABN_, CV_TSN_, CV_FSN_	9	133,977,561	-	U6/-
H3GA0029253	FSN	10	11,009,092	-	DUSP10/HHIPL2
ASGA0101355	MOT	10	15,906,379	-	PLD5/HHIPL2
WU_10.2_10_65681460	CV_DEN_, CV_MOT_, CV_TSN_, CV_FSN_	10	59,980,174	-	UPF2/PROSER2
MARC0097207	CV_DEN_, CV_MOT_, CV_ABN_, CV_TSN_, CV_FSN_	12	16,290,215	-	TLK2/EFCAB3
MARC0113309	CV_DEN_, CV_MOT_, CV_ABN_, CV_TSN_, CV_FSN_	12	22,708,807	-	PPP1R1B/NEUROD2
ASGA0058857	CV_VOL_, CV_MOT_	13	132,659,741	-	BDH1/DLG1
ALGA0077301	CV_MOT_, CV_FSN_	14	44,439,785	-	CRYBA4/-
ASGA0063383	CV_FSN_	14	50,930,578	DGCR2	ZNF74/TSSK1B
ASGA0063433	CV_VOL_	14	53,990,384	RYR2	ZP4/-
DRGA0013964	CV_DEN_, CV_MOT_, CV_TSN_, CV_FSN_	14	63,781,108	-	U6/-
WU_10.2_14_135493445	CV_DEN_, CV_MOT_, CV_TSN_	14	124,371,607	-	NHLRC2/ADRB1
WU_10.2_16_238186	CV_MOT_, CV_ABN_, CV_FSN_	16	514,821	CTNND2	DAP/U6
WU_10.2_17_4199456	CV_ABN_, CV_FSN_	17	3,947,416	-	MSR1/-
WU_10.2_17_5093088	CV_DEN_, CV_TSN_	17	4,699,290	FGF20	U2/MICU3
ALGA0110656	CV_DEN_, CV_ABN_, CV_TSN_, CV_FSN_	17	4,872,377	ZDHHC2	MICU3/CNOT7
WU_10.2_17_69108942	CV_VOL_	17	61,588,040	SS18L1	PSMA7/MTG2
MARC0056921	CV_FSN_	18	6,400,611	-	GIMAP2/GIMAP4
WU_10.2_18_13174146	CV_ABN_	18	12,468,287	CHRM2	Metazoa_SRP/ssc-mir-490-1
WU_10.2_18_15516325	CV_VOL_	18	14,653,756	-	BPGM/AKR1B1
Affx-115137014	CV_ABN_	18	26,996,757	-	U2/-
ASGA0080065	CV_VOL_, CV_DEN_, CV_MOT_, CV_ABN_, CV_TSN_, CV_FSN_	18	45,424,614	HOXA5	HOXA7/HOXA3

## Data Availability

The datasets used or analyzed during the present study are available from the corresponding author upon reasonable request.

## Supplementary Materials

The following supporting information can be downloaded at: https://www.mdpi.com/article/10.3390/ani14131839/s1, Figure S1: The number of observations per each fixed-effect-level; Table S1: DEBV of semen traits; Table S2: Estimates of heritability for semen traits in pigs.
