# Supplementary material for: Genome-Wide Association Study Reveals Novel Candidate Genes Influencing Semen Traits in Landrace Pigs
Source: Animals (Basel). 2024 Jun 21;14(13):1839. doi: 10.3390/ani14131839 (PMC11240458; doi:10.3390/ani14131839)
Supplement: Supplementary file 1 [file animals-14-01839-s001.zip › animals-3016744-supplementary/Table S2.docx]

**Table S2 Estimates of heritability for semen traits in pigs**

| Traits | a | pe | e | *h*2 |
| --- | --- | --- | --- | --- |
| VOL | 1178.756 | 1153.563 | 3560.375 | 0.20±0.02 |
| DEN | 0.414 | 0.504 | 1.582 | 0.17±0.05 |
| MOT | 16.216 | 19.564 | 35.13 | 0.23±0.03 |
| ABN | 12.067 | 14.832 | 23.56 | 0.24±0.03 |
| TSN | 8267.954 | 16514.494 | 49197.668 | 0.11±0.02 |
| FSN | 10278.591 | 13664.336 | 42591.089 | 0.15±0.02 |
| CV_VOL_ | 1.762 | 21.036 | 80.591 | 0.017±0.009 |
| CV_DEN_ | 0.533 | 30.783 | 157.734 | 0.003±0.005 |
| CV_MOT_ | 0.572 | 129.239 | 380.513 | 0.001±0.006 |
| CV_ABN_ | 4.353 | 32.989 | 341.521 | 0.011±0.006 |
| CV_TSN_ | 1.716 | 76.357 | 904.451 | 0.002±0.004 |
| CV_FSN_ | 11.652 | 61.423 | 191.047 | 0.044±0.014 |
