# Supplementary material for: Genome-Wide Association Study Reveals Novel Candidate Genes Influencing Semen Traits in Landrace Pigs
Source: Animals (Basel). 2024 Jun 21;14(13):1839. doi: 10.3390/ani14131839 (PMC11240458; doi:10.3390/ani14131839)
Supplement: Supplementary file 1 [file animals-14-01839-s001.zip › animals-3016744-supplementary/Table S1.docx]

**Table S1 DEBV of semen traits**

| Traits | DEBV | | | | REL |
| --- | --- | --- | --- | --- | --- |
|  | MEAN | STD | MAX | MIN |  |
| VOL | 0.19 | 10.58 | 38.02 | -36.02 | 0.56 |
| DEN | -0.02 | 0.19 | 0.74 | -0.78 | 0.55 |
| MOT | 0.03 | 0.60 | 2.89 | -2.16 | 0.48 |
| ABN | -0.05 | 2.02 | 10.94 | -6.37 | 0.60 |
| TSN | 0.39 | 13.66 | 47.21 | -59.19 | 0.43 |
| FSN | 0.49 | 19.51 | 76.99 | -86.36 | 0.57 |
| CV_VOL_ | 3.81 | 204.65 | 12162.21 | -519.58 | 0.55 |
| CV_DEN_ | -3.04 | 72.34 | 94.21 | -2623.36 | 0.54 |
| CV_MOT_ | -3.71 | 69.18 | 75.94 | -2529.59 | 0.59 |
| CV_ABN_ | -1.08 | 35.48 | 49.98 | -1586.68 | 0.61 |
| CV_TSN_ | -7.42 | 181.32 | 86.88 | -6375.87 | 0.44 |
| CV_FSN_ | -12.73 | 330.83 | 81.86 | -11108.32 | 0.48 |
