# Supplementary material for: Genome-Wide Association Study Reveals Novel Candidate Genes Influencing Semen Traits in Landrace Pigs
Source: Animals (Basel). 2024 Jun 21;14(13):1839. doi: 10.3390/ani14131839 (PMC11240458; doi:10.3390/ani14131839)
Supplement: Supplementary file 1 [file animals-14-01839-s001.zip › animals-3016744-supplementary/Figure S1.docx]

**a**

**
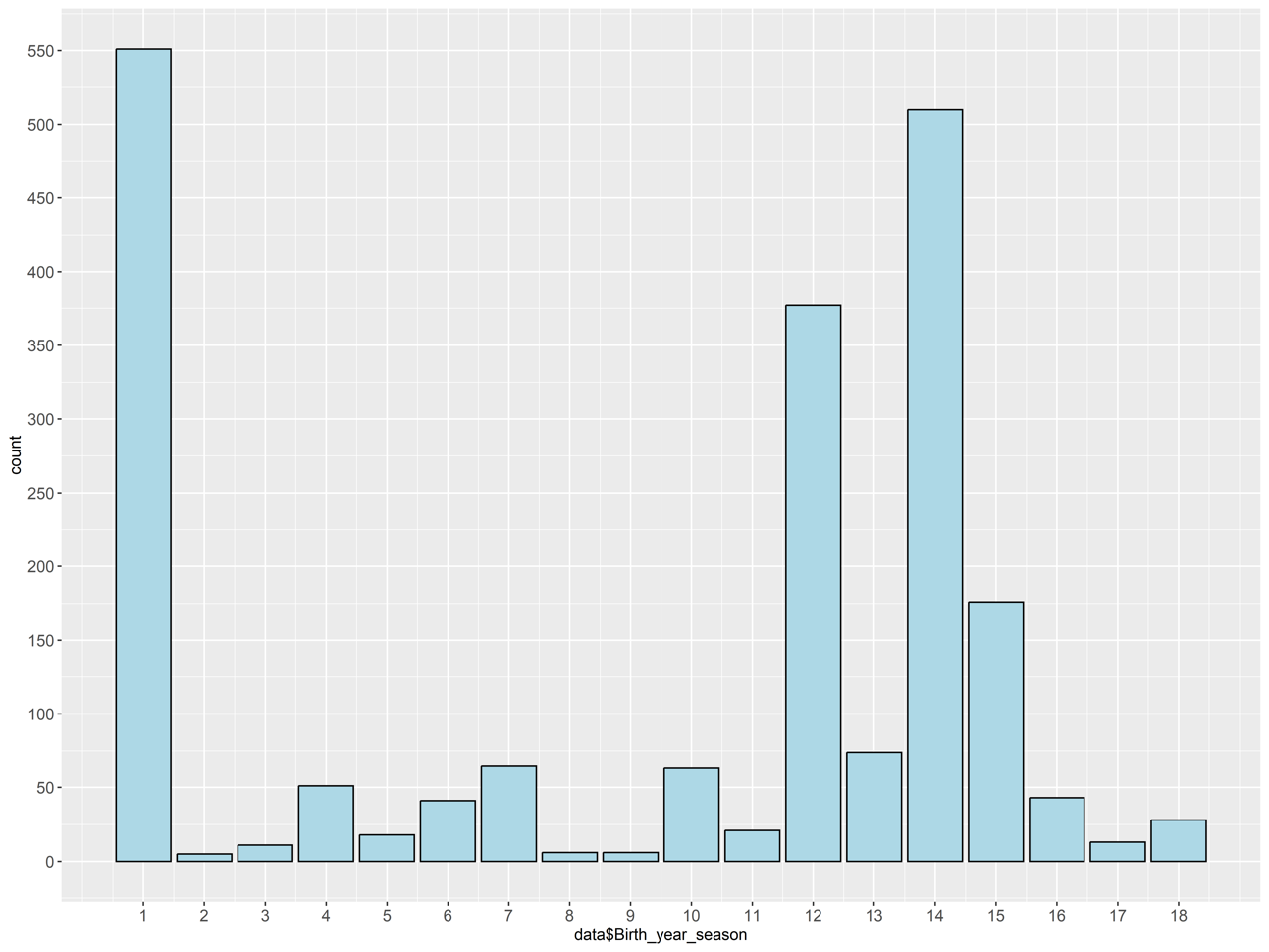
**

**b**

**
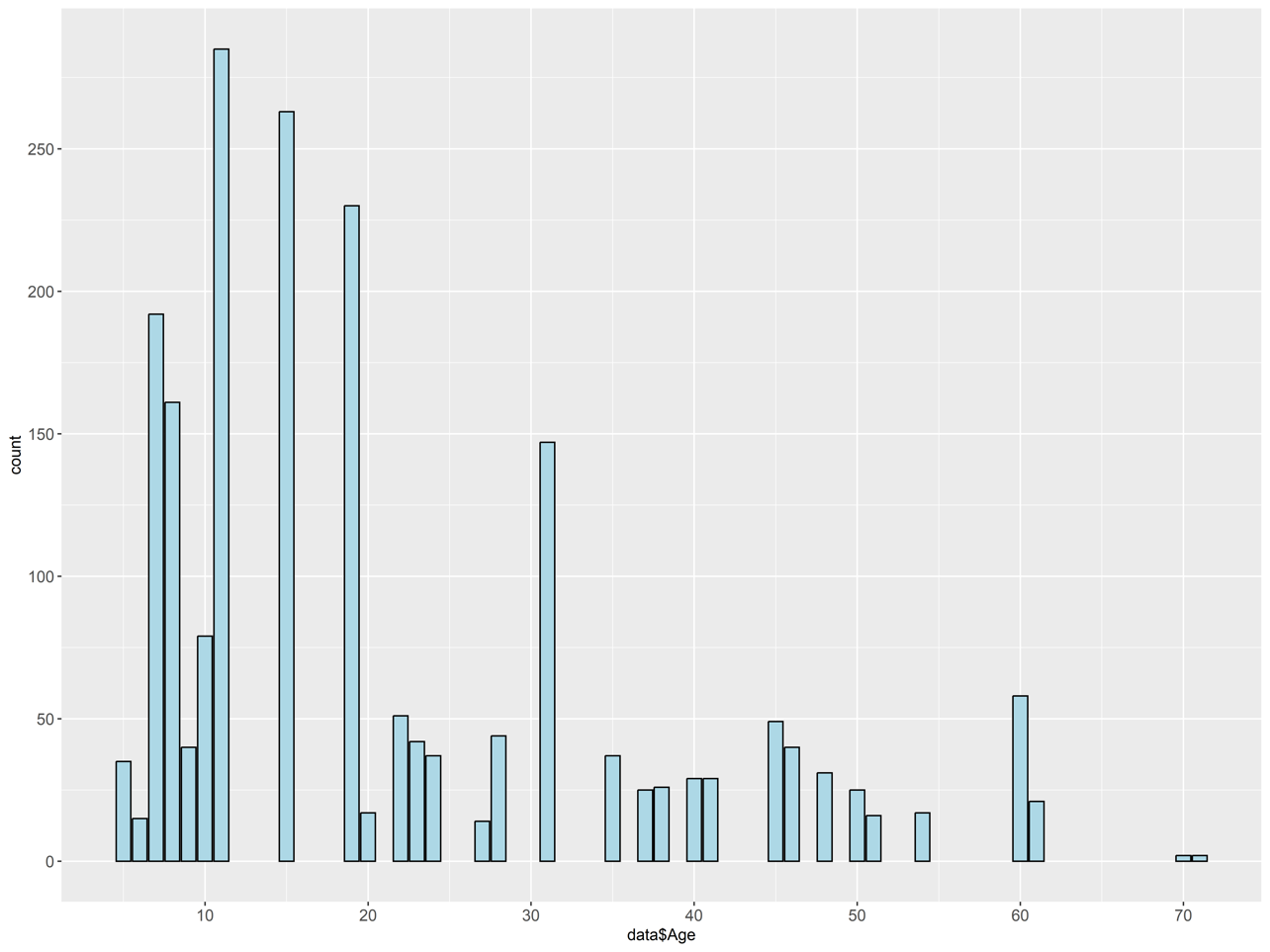
**

**c**

**
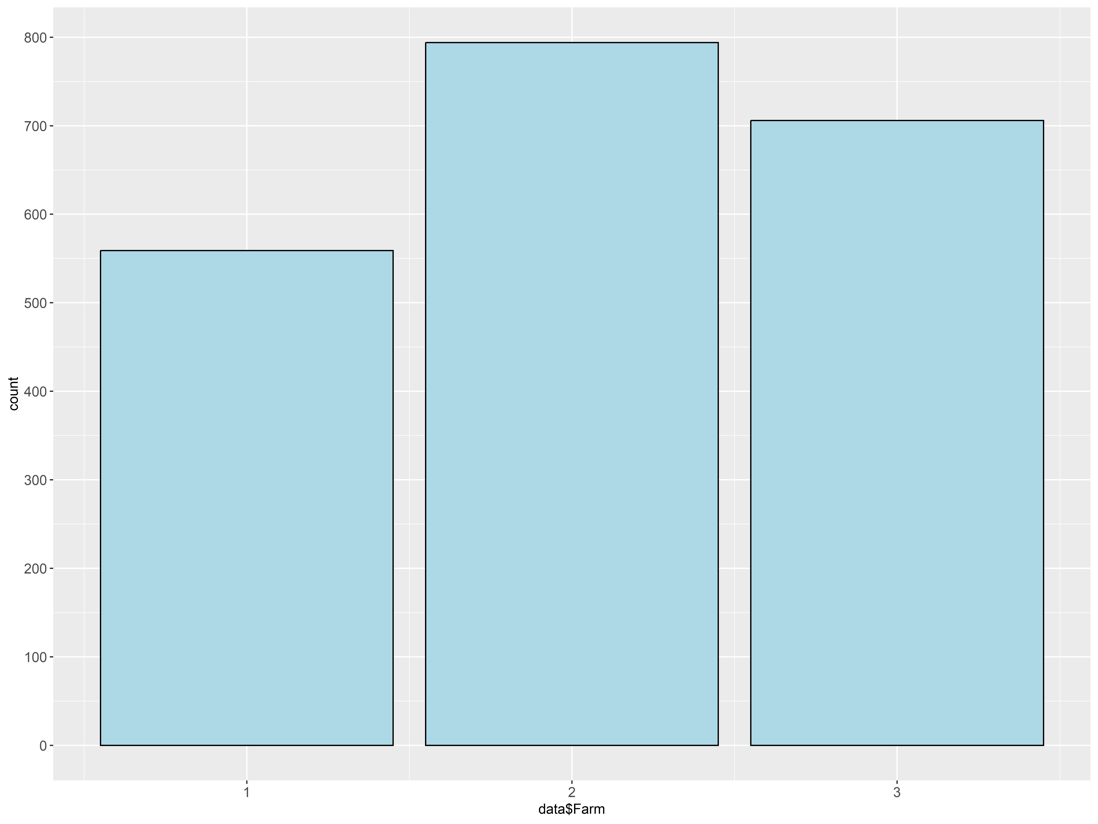
**

**Figure S1 The** **number of observations per each fixed-effect-level. a** Birth year-season (levels: 18); **b** Age (month, levels: 31); **c** Farm (levels: 3).
